# Validity of pervasive computing based continuous physical activity assessment in community-dwelling old and oldest-old

**DOI:** 10.1038/s41598-019-45733-8

**Published:** 2019-07-04

**Authors:** Narayan Schütz, Hugo Saner, Beatrice Rudin, Angela Botros, Bruno Pais, Valérie Santschi, Philipp Buluschek, Daniel Gatica-Perez, Prabitha Urwyler, Laura Marchal-Crespo, René M. Müri, Tobias Nef

**Affiliations:** 1 Gerontechnology & Rehabilitation Group, University of Bern, Bern, Switzerland; 2 Department of Cardiology, University Hospital Bern (Inselspital), University of Bern, Bern, Switzerland; 3 Department of Neurology, University Neurorehabilitation Unit, University Hospital Bern (Inselspital), University of Bern, Bern, Switzerland; 4 Höhere Fachschule Pflege, Berufsbildungszentrum Olten, Olten, Switzerland; 5 La Source, School of Nursing Sciences, HES-SO University of Applied Sciences and Arts Western Switzerland, Lausanne, Switzerland; 6 DomoSafety S.A., Lausanne, Switzerland; 7 Idiap Research Institute, Martigny, Switzerland; 8 ARTORG Center for Biomedical Engineering Research, University of Bern, Bern, Switzerland; 9 École Polytechnique Fédérale de Lausanne (EPFL), Lausanne, Switzerland; 10 Sensory‐Motor Systems (SMS) Lab, Institute of Robotics and Intelligent Systems (IRIS), Department of Health Sciences and Technology (D‐HEST), ETH Zürich, Zürich, Switzerland

**Keywords:** Geriatrics, Biomedical engineering, Ageing

## Abstract

In older adults, physical activity is crucial for healthy aging and associated with numerous health indicators and outcomes. Regular assessments of physical activity can help detect early health-related changes and manage physical activity targeted interventions. The quantification of physical activity, however, is difficult as commonly used self-reported measures are biased and rather unprecise point in time measurements. Modern alternatives are commonly based on wearable technologies which are accurate but suffer from usability and compliance issues. In this study, we assessed the potential of an unobtrusive ambient-sensor based system for continuous, long-term physical activity quantification. Towards this goal, we analysed one year of longitudinal sensor- and medical-records stemming from thirteen community-dwelling old and oldest old subjects. Based on the sensor data the daily number of room-transitions as well as the raw sensor activity were calculated. We did find the number of room-transitions, and to some degree also the raw sensor activity, to capture numerous known associations of physical activity with cognitive, well-being and motor health indicators and outcomes. The results of this study indicate that such low-cost unobtrusive ambient-sensor systems can provide an adequate approximation of older adults’ overall physical activity, sufficient to capture relevant associations with health indicators and outcomes.

## Introduction

It is commonly known and widely accepted that physical activity positively influences health. There is strong scientific evidence that physical activity reduces the risk for a variety of health outcomes like high blood pressure, type 2 diabetes, cancer, weight gain, falls, depression, loss of cognitive function or functional ability in seniors^1,2^. While these findings are of high relevance for all age groups, they are of special importance for the growing number of old and even more so for the oldest-old adults – especially since physical activity is a modifiable risk factor^3,4^. In addition, seniors are more likely to suffer from chronic diseases, experience falls or face significant cognitive decline. They are also more prone to a sedentary lifestyle^5^ and results of cardiorespiratory fitness measures even suggest an age-related acceleration in decline^6^, which might also be detectable by physical activity.

While it is evident that moderate-to-vigorous-intensity physical activity is usually better, research suggests that light- and moderate-intensity physical activity is still better than no physical activity in terms of health benefits^2^. This is important for seniors as they may often find it difficult to engage in high-intensity physical activities such as running or aerobic exercise. Light- and moderate-intensity physical activities like cooking, vacuuming or other everyday activities, constitute an important and often integral part in older adult’s total physical activity. Measuring this type of physical activity is rather difficult but may be very important for the early detection of preventable physical activity decline or to monitor the course of interventions. Today, physical activity assessments are often based on self-reporting which is not only prone to response bias but also suffers from recall bias – especially with declining memory^4,7–9^. Frequently used alternatives are accelerometer or pedometer based^7,10^. While these provide objective physical activity measures in free-living conditions, they must be worn, which becomes cumbersome in long-term assessments of several months or even years and is thus often accompanied by wear-time dependent non-compliance issues^10^.

Advances in technology made pervasive computing feasible for technology assisted healthy aging by embedding smart microprocessor-driven computing devices in everyday objects (as for instance seen in appliances of smart homes)^11^. A growing body of groundbreaking research shows that such systems are not only feasible and well accepted by seniors but are also useful for the detection of emergency situations or early changes in health status^9,12,13^. A frequently used and increasingly commercialized technology is passive infrared (PIR) motion sensing, which is both inexpensive and unobtrusive, to an extent that people tend to forget about it^14,15^. In this context, PIR motion sensors work by detecting the presence of a person’s motion in an equipped room^16^. Besides safety applications^17–20^, most work in this direction primarily targeted cognitive outcomes. Galambos *et al*. for instance showed that changes in PIR-sensor derived motion density maps correspond to exacerbations of depression and dementia^21^. In a similar manner Hayes *et al*. demonstrated that variability in PIR-sensor derived activity and gait-speed data differed between cognitively normal subjects and those with mild cognitive impairment (MCI)^22^. Similarly, Urwyler *et al*. highlighted the difference between sensor derived activities of daily living patterns in healthy and MCI subjects^23^.

In this work, we assess the potential of PIR-sensors in the light of physical activity. In particular, we explore the validity and potential of unobtrusive, continuous PIR-sensor readings for physical activity quantification, targeting in-home light- and moderate-intensity physical activity. Towards this goal, we analyzed the behavior of PIR-sensor based (physical) activity metrics and compared them with a multitude of cognitive, well-being and motor-function related assessments to see whether this approximation to physical activity sufficiently captures known effects of physical activity on commonly used health indicators and outcomes. The data for the analysis stems from a naturalistic sample of thirteen community dwelling old and oldest-old Swiss subjects (age = 90.9 ± 4.3 years, female = 69.23%) from the StrongAge cohort in Olten (Switzerland). All analyzed subjects shared the same apartment layout. The subjects were monitored for the duration of one year. Simultaneously, a battery of standardized clinical tests and assessments were performed repeatedly. The resulting data was aggregated and analyzed in terms of baseline differences. In addition, physical activity data from a subject with rapid health decline was evaluated and visualized in a case study format.

## Results

**Figure 1:**
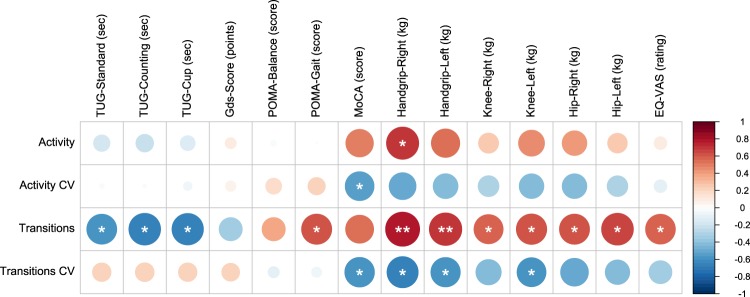
Visual Correlation Matrix of the four sensor-derived physical activity metrics and the clinical assessments). Shown is a visual representation^h^ of the respective correlations as measured by the Spearman’s rank correlation coefficients (ρ) based on an α = 0.05^i^. The sensor-derived physical activity metrics (rows) represent the mean and the coefficient of variation (CV) of the daily measurements over the whole monitoring duration. The size as well as colour-intensity signal the correlation strength, where red means a strong positive and blue a strong negative correlation. ^a^Timed Up & Go (TUG)^27^ (Counting = while additionally counting backwards from 100; Cup = while holding a full cup of water). ^b^Geriatric Depression Scale (GDS)^25^. ^c^Tinetti Performance-oriented mobility assessment (POMA)^28^. ^d^Montreal cognitive assessment (MoCA)^24^. ^e^Knee extensor strength (Knee). ^f^Hip flexor strength (Hip). ^g^Visual analogue scale: measuring perceived health based on the EQ-5D-3L system (EQ-VAS)^26^. ^h^created using the R package “corrplot”^34^ ^i^*<0.05; **<0.01.

Over roughly one year, more than 89’389 person-hours were recorded from the homes of thirteen old and oldest-old participants (age = 90.9 ± 4.3 years) (Table 1), all sharing the same apartment layout and sensor placement. During the same period, classic assessments of multiple health outcomes have been assessed. Two normalized PIR-sensor derived measures of physical activity were calculated. First, the daily sensor activity – measuring the time the sensors were detecting activity (Equation (1)). Second, the normalized daily number of room-transitions (measuring the hourly number of transitions between different rooms) (Equation (2)). Here, we present the resulting associations and observations between these sensor-based physical activity metrics and the classic clinical assessments (Fig. 1).

**Table 1:** Participant characteristics and demographics.

Participant	Age (years)	Sex	BMI (kg/m^2^)	MoCA^a^	Hours Monitored
1	94	f	21.502	15	6925.428
2	91	m	25.911	23	7594.068
3	95	f	24.006	25	7595.858
4	91	m	25.952	25	7596.852
5	88	m	27.465	24	7596.960
6	91	m	22.309	14	7597.152
7	94	f	28.076	25	7595.049
8	80	f	24.387	21	7299.515
9	89	f	23.336	22	7587.843
10	94	f	27.344	18	7595.700
11	98	f	22.491	23	3744.691
12	89	f	23.795	14	7597.119
13	88	f	22.269	17	3062.842

^a^At inclusion.

### Cognitive function and well-being

With regard to cognitive and well-being factors, three different assessments were analysed: the Montreal Cognitive Assessment (MoCA)^24^, the Geriatric Depression Scale (GDS)^25^ and the EQ-VAS score (EQ-VAS as part of the EQ-5D-3L)^26^. EQ-VAS scores showed a significant correlation with the number of room-transitions (ρ = 0.593, p = 0.033). However, no associations with depression were found – as measured by the GDS. General cognitive functioning, as measured by the MoCA, was negatively correlated with the coefficient of variation (CV) of the sensor activity (ρ = −0.556, p = 0.048) as well as with the CV of the number of room-transitions (ρ = −0.587, p = 0.035).

### Motor function

Multiple motor function related factors, consisting of muscle strength (handgrip, knee extensor and hip flexor) as well as mobility measures, were examined. Mobility measures included the fall risk related timed up and go (TUG)^27^ test and the balance and gait focused Tinetti performance-oriented mobility assessment (POMA)^28^.

Amongst the measured muscle groups, right hand handgrip strength showed the strongest correlation with both physical activity measures (sensor activity: ρ = 0.692, p = 0.009; number room-transitions: ρ = 0.775, p = 0.002). The remaining muscle groups were only correlated to the number of room-transitions metric and apart from the right knee and right hip also to the CV of the number of room-transitions (handgrip right: ρ = −0.648, p = 0.017 and handgrip left: ρ = −0.577, p = 0.039).

Concerning the TUG times, the cognitive variant (walking while simultaneously counting backwards) had the strongest negative correlation with the number of room-transitions metric (ρ = −0.670, p = 0.012), but also the times for the normal and manual TUG variant showed significant negative correlations with the number of room-transitions (ρ = −0.599, p = 0.031 and ρ = −0.659, p = 0.014, respectively). The POMA score for gait showed a negative correlation with the number of room-transitions (ρ = 0.606, p = 0.028) but no significant correlation was found in case of the POMA balance score.

### Case study of a subject with a rapid decline in health

**Figure 2:**
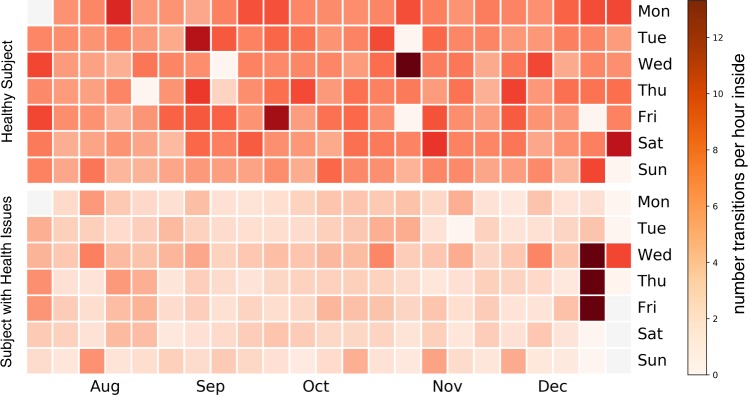
Room-transition heatmaps: comparison between healthy subject and subject with health issues. Shows two heatmaps comparing five months of physical activity measured by the number of room-transitions. One example of a healthy participant (participant 9) with a rather active lifestyle (upper) and the other one of a subject (participant 11) which developed severe and eventually fatal health issues (lower). Note the increased number of transitions throughout the second last week of the subject with health issues, distinctly showing the influence of visits from nurses, family and friends. (more intense colour signifies more room-transitions).

**Figure 3:**
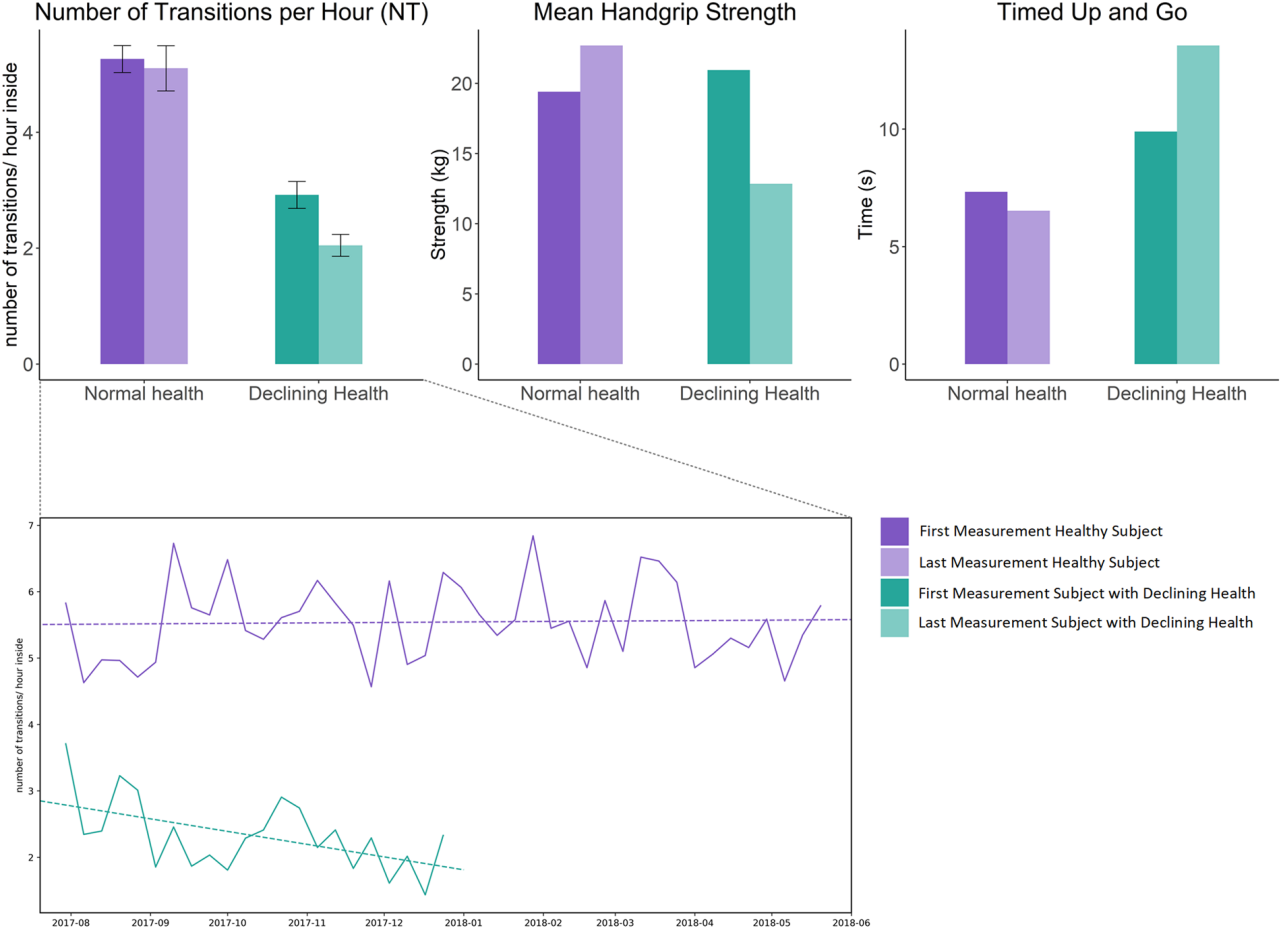
Comparison of first and last measurements of multiple assessments in a healthy subject and one with rapidly declining health. Shows a case of rapid declining health (participant 11) and compares it with data from a healthy reference subject (participant 9). As such, the average in room-transitions of the first and last recorded month is displayed (left). In a similar manner, the handgrip strength (middle) and timed up and go (TUG) times (right) of the first and last assessments are shown. In all cases, for the subject with health issues there was a decrease in metrics (less room-transitions, less handgrip-strength, longer TUG times), while the healthy subject did not exhibit negative changes.

Although one year is rather short to capture significant health changes in such a small population sample, the relationship between health and our physical activity metrics can be shown visually in one participant (participant 11) with a very quick and eventually fatal decline in health. In that regard we visualized the course in room-transition based physical activity between a healthy subject (participant 9) and the one with significant health issues (Figs 2 and 3). It is apparent that not only did the participant with health issues exhibit a more sedentary lifestyle to begin with (visible in the difference of base levels in physical activity) but also did the measured physical activity decrease in a short time-frame.

## Discussion

To evaluate the feasibility and validity of PIR-sensor based physical activity assessments, we analysed the relationship of sensor derived physical activity metrics with results from standardized clinical assessments. The results from thirteen community-dwelling seniors allowed us to evaluate whether this approach towards physical activity quantification captures similar relationships with well-being, cognitive and motor function as conventional physical activity itself. The main advantage of PIR-sensor based physical activity measurements over traditional methods is its ability to objectively, continuously and unobtrusively measure light- and moderate-intensity physical activity. This might allow for gapless longitudinal assessment of physical activity over the course of years and maybe even decades, which could benefit from early detection of physical activity decline (and subsequently to a reasonable degree also general health) and improve management of respective interventions^7^. In addition, it might also facilitate physical activity research in older adults.

### Clinical assessments

Fall risk, estimated by TUG times, is negatively correlated to baseline values of room-transitions, indicating that more room-transitions reduce fall risk. Similarly, gait performance was positively correlated with the room-transitions – as measure by the gait score of the POMA. Muscle strength measures were predominantly correlated to room-transitions, with the exception of right-hand grip strength, which was also found to correlate with sensor activity. So far, these findings are in line with research about physical activity^1,2^. Interestingly, depression (measured by the GDS) had no significant correlations although literature would suggest otherwise^2^. While this could probably be explained by the small sample size and a rather optimistic study population, it might also be related to the measured intensity of physical activity. Many depression related physiological benefits of physical activity are primarily related to higher intensity physical activity^29^. Similarly to the GDS, MoCA derived cognitive functioning was not correlated to either of the sensor-derived metrics, in contrast to what physical activity literature would state. However, the CV of sensor activity and room-transitions showed a strong relationship with MoCA scores, which supports multiple findings, showing increased variance in the behavior of people with MCI^21–23^. Although highly speculative, this could suggest that the variation in daily physical activity levels is an even more important hallmark of cognitive decline than low baseline physical activity levels. Self-rated health quantified by the (EQ-VAS) did show a significant correlation with the number of room-transitions which reflects findings about health-related quality of life and physical activity^30,31^.

### Sensor-derived metrics

Overall, the number of room-transitions metric was much stronger and more frequently correlated to clinical assessment results, when compared to the sensor activity metric. A possible explanation would be that the number of room-transitions represents a higher level of physical activity than sensor activity does. This seems plausible since transitions between rooms require a person to be at least walking, while sensor activity could also be largely generated due to light-intensity physical activity. Another reason might be that the number of room-transitions is just better comparable (less variation due to noise) between different subjects – since all share the same apartment layouts, a transition means mostly the same movement, irrespective of the person, while activity may be influenced by factors like the location and consequentially the distance and angle to the sensor. Concerning literature, the limited body of research about the usefulness of different PIR-sensor based metrics is inconclusive. While a case study from Campbell *et al*. suggested that the daily number of transitions could be useful in detecting changes in health status^32^, other studies made similar claims about activity^33^ – for the sake of simplicity, we here refer to the number of sensor firings and sensor activity as the same.

### Case study

Retrospective findings from a case study of a senior with rapid declining health (participant 11), which eventually led to the senior’s death, showed a visible and rapid decline in measured physical activity and repeated clinical assessments, including TUG times and muscle strength (Fig. 3). This is while all three measures remained approximately steady in case of the reference subject (participant 9). It is also noticeable that baseline physical activity of this participant was already low at baseline, when compared to a healthy reference (see Figs 2, 3). It is even possible that the PIR-sensor based physical activity decline would have been more drastic if the growing number of visits from nurses, family and friends (notice the dark red days throughout the second last week of December in Fig. 2) were completely excluded from the data. These results further confirm the intuitive assumption that fast changes in physical activity can be measured using PIR-sensor based physical activity metrics and that these changes may be a response to changes in overall health, which further validates similar findings from other studies^12,32,33^.

### Limitations

One of the main limitations of PIR-sensor derived physical activity is the fact that it can only measure in-home physical activity, which may not show the whole range of physical activity a senior engages in. In addition, baseline physical activity evaluations, thus inter-individual comparisons will be difficult to extend to older adults with different apartment layouts. Note that this does not affect intra-individual physical activity changes and patient specific characteristics. However, based on our observations intra-individual change, if not induced by short-term illness (as for instance in the highlighted case study) has high variability and potential seasonal patterns, likely requiring data over multiple years to quantify less significant trends. Another limitation is that we cannot currently distinguish between multiple persons in the apartment, thus the method is only applicable for seniors living alone and who do not have frequent long-term visits that would significantly offset sensor readings. It is further not clear how the results would apply to similar aged populations with different local culture as the main assumption of this approach is based on the observation that Central European seniors spend a very significant amount of time inside their homes.

### Outlook

Future research with different senior populations will be necessary to validate the proposed physical activity assessment method and how it is related to health. In addition, it will be very important to extend the monitoring duration to several years to exclude seasonal trends and to better quantify the effect of the weaker intra-individual changes, instead of just baseline differences. Especially for potential clinical applications it would be important to validate individual changes in a larger population to identify threshold values which signify a specific risk of a health state change. To further validate this approach, it might also be important to compare the physical activity measured by PIR-sensors with simultaneously recorded data from accelerometers or pedometers.

## Conclusion

To sum up, we found that PIR-sensor based metrics of physical activity, especially the number of room-transitions, to be associated with well-being as well as cognitive and motor function. These findings are in agreement with literature analyzing the effects of physical activity on health indicators and outcomes^2^. Therefore, we conclude that the PIR-sensor derived number-room transitions metric serves as a sufficient approximation of the true physical activity in community-dwelling Swiss seniors. Findings from a case study and related findings from other studies that employed similar sensor setups^12,32,33^ further confirm such a link between PIR-sensor measures and various health indicators and outcomes. Thus, PIR-sensor based assessment of physical activity could be a cost-effective and plausible approach for continuous, objective and unobtrusive long-term assessment of light- and moderate-intensity physical activity, which avoids the downsides of commonly used methods and bears the potential to aid in technology assisted healthy-aging.

## Methods

### Participants

The data presented here stems from a study where thirteen Swiss, community dwelling seniors, were equipped with pervasive computing systems for approximately one year. Inclusion criteria were based on age (≥80 years), the ability to live in an own apartment or house and to live alone. Recruitment aimed at representing a naturalistic sample of alone living, community dwelling older adults in central Switzerland, irrespective of their cognitive status.

The related study was conducted based on principles declared in the Declaration of Helsinki and approved by the Ethics Committee of the canton of Bern, Switzerland (KEK-ID: 2016-00406). All subjects signed and handed in an informed consent before study participation.

### Clinical assessments

Clinical assessments were conducted at the beginning of the study and consisted of a battery of standardized tests, targeting well-being, cognitive and motor function. The cognitive and well-being part included the Montreal Cognitive Assessment (MoCA), the Geriatric Depression Scale (GDS) as well as EQ-5D-3L. The motor tests included the Tinetti Performance-Oriented Mobility Assessment (POMA), the Timed Up and Go (TUG) as well as muscle strength measurements for handgrip, knee extensor and hip flexor – for all three muscle-groups, the left and right-side strength was measured. The handgrip measurements were performed using a Jamar Plus + Dynamometer while knee and hip strength was assessed with a Lafayette® Manual Muscle Tester (Lafayette Instrument Company, Lafayette, Indiana).

In addition to the initial assessment, muscle strength and TUG measures were repeated every 6th week and where possible, the whole initial battery was repeated after one year (a different variation of the MoCA was used there to avoid memory effects). Throughout the whole study duration, the subjects were visited or contacted on a weekly basis to stay informed about sudden changes in health or lifestyle. As part of these visits, the participants were asked to fill out EQ-5D-3L questionnaires, including EQ-VAS scores.

More information regarding subject demographics and characteristics is summarized in Table 1.

### Sensor setup

**Figure 4:**
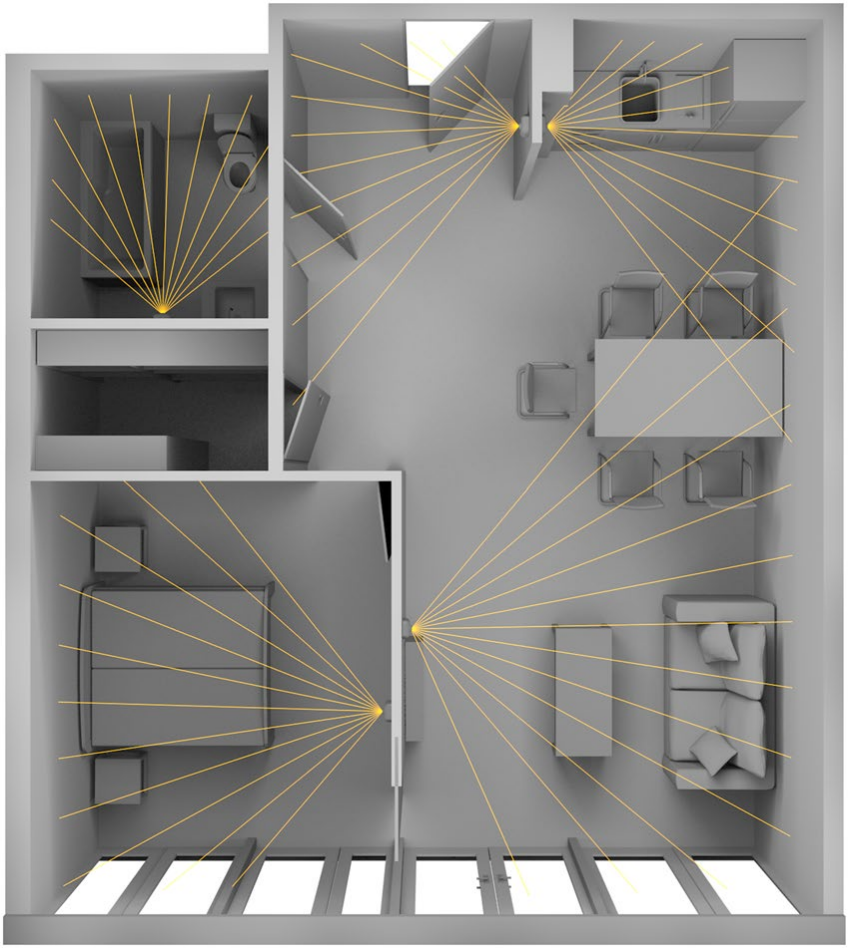
Exemplary apartment layout and PIR-sensor placement. Gives a broad idea of the kind of apartments we monitored and where the sensors where placed.

The presented data was obtained using the commercial DomoCare® home monitoring system for seniors (DomoSafety S.A., Lausanne, Switzerland)^15^. This system included five passive infrared (PIR) motion sensing units and two magnetic door sensors that communicate with a base unit via the Zigbee protocol. The motion sensors measure presence or absence of motion once every two seconds (0.5 Hz). The base unit manages the data and sends it to the cloud in real-time using the GSM network. The subject’s kitchen, living room, entrance, bedroom and bathroom were each equipped with one PIR-sensor (see Fig. 4).

The two door sensors were placed at the fridge and entrance doors. Wherever possible, the sensors were placed at the exact same locations in each apartment. Due to furniture related constraints some placements did vary slightly but were kept as comparable as possible.

### Data analysis

The clinical tests and muscle measurements were aggregated by averaging, where not otherwise mentioned. The sensor data was first pre-processed to remove days with extremely high or low activity (based on the 1st and 99th percentile of a maximum likelihood fitted normal distribution). Subsequently the activity-metrics for sensor activity and the number of room-transitions were calculated daily, for each participant:

1 $$\begin{array}{ccc}{\hat{\mu }}_{i}^{ac} & = & \frac{1}{{n}_{days}}{\sum }_{j=1}^{{n}_{days}}{{\rm{s}}{\rm{e}}{\rm{n}}{\rm{s}}{\rm{o}}{\rm{r}}{\rm{\_}}{\rm{a}}{\rm{c}}{\rm{t}}{\rm{i}}{\rm{v}}{\rm{i}}{\rm{t}}{\rm{y}}}_{j}\\ {{\rm{s}}{\rm{e}}{\rm{n}}{\rm{s}}{\rm{o}}{\rm{r}}{\rm{\_}}{\rm{a}}{\rm{c}}{\rm{t}}{\rm{i}}{\rm{v}}{\rm{i}}{\rm{t}}{\rm{y}}}_{j} & = & \frac{{t}_{motion}}{{t}_{inside}}\,\ast \,100,\end{array}$$

2 $$\begin{array}{ccc}{\hat{\mu }}_{i}^{tr} & = & {\sum }_{j=1}^{{n}_{days}}\,{\rm{n}}{\rm{u}}{\rm{m}}{\rm{b}}{\rm{e}}{\rm{r}}\,{\rm{o}}{\rm{f}}\,{\rm{r}}{\rm{o}}{\rm{o}}{\rm{m}}\,{\rm{t}}{\rm{r}}{\rm{a}}{\rm{n}}{\rm{s}}{\rm{i}}{\rm{t}}{\rm{i}}{\rm{o}}{\rm{n}}{\rm{s}}\,{\rm{p}}{\rm{e}}{\rm{r}}\,{{\rm{h}}{\rm{o}}{\rm{u}}{\rm{r}}}_{j}\\ {\rm{n}}{\rm{u}}{\rm{m}}{\rm{b}}{\rm{e}}{\rm{r}}\,{\rm{o}}{\rm{f}}\,{\rm{r}}{\rm{o}}{\rm{o}}{\rm{m}}\,{\rm{t}}{\rm{r}}{\rm{a}}{\rm{n}}{\rm{s}}{\rm{i}}{\rm{t}}{\rm{i}}{\rm{o}}{\rm{n}}{\rm{s}}\,{\rm{p}}{\rm{e}}{\rm{r}}\,{{\rm{h}}{\rm{o}}{\rm{u}}{\rm{r}}}_{j} & = & \frac{{\rm{\#}}tr}{{t}_{inside}\div3600\,\frac{s}{h}},\end{array}$$

Here *n*_*days*_ refers to a subject’s number of recorded days, while *j* references a specific day. The parameter *t*_*motion*_ represents the number of seconds where the PIR sensors detected motion, *t*_*inside*_ represents the number of seconds a person was at home for a given day *j* and #*tr* represents the number a person transitioned between the different rooms of the apartment, as measured by the PIR sensors per day. For comparison of baseline differences, the sample means across all included days (${\hat{\mu }}_{i}$) of subject *i* for both metrics were calculated. The coefficient of variations, *CV*_*i*_, of subject *i* was derived by dividing the sample standard deviation ${\hat{\sigma }}_{i}$ by the sample mean ${\hat{\mu }}_{i}$ of the respective physical activity metric.

$$C{V}_{i}=\frac{{\hat{\sigma }}_{i}}{{\hat{\mu }}_{i}}$$

$${\hat{\mu }}_{i}=\frac{1}{m}\sum _{k}^{m}{\hat{\mu }}_{k}$$

$${\hat{\sigma }}_{i}=\sqrt{\frac{1}{m-1}\sum _{j}^{{n}_{days}}{({\hat{\mu }}_{j}-{\hat{\mu }}_{i})}^{2}}$$

To calculate the correlations between the assessments and the activity metrics (matrix ***R***, as visualized in Fig. 1), the nonparametric Spearman’s rank correlation coefficient *ρ*_*kl*_ between mean aggregated assessment results (*C*_*l*_) and average activity metrics (***A***_*k*_) was used. Here, *C*_*l*_ and ***A***_*k*_ denote the *l*^*th*^ and *k*^*th*^ column of matrix ***C*** and ***A***, respectively.

$$\begin{array}{c}{({\boldsymbol{R}})}_{kl}={{\boldsymbol{\rho }}}_{kl}=Corr({{\boldsymbol{A}}}_{k},{{\boldsymbol{C}}}_{l}),{\boldsymbol{R}}\in {{\mathbb{R}}}^{4\times 14}\\ {\boldsymbol{A}}=[\begin{array}{cccc}{\hat{\mu }}_{1}^{ac} & C{V}_{1}^{ac} & {\hat{\mu }}_{1}^{tr} & C{V}_{1}^{tr}\\ \vdots  & \vdots  & \vdots  & \vdots \\ {\hat{\mu }}_{i}^{ac} & C{V}_{i}^{ac} & {\hat{\mu }}_{i}^{tr} & C{V}_{i}^{tr}\\ \vdots  & \vdots  & \vdots  & \vdots \\ {\hat{\mu }}_{M}^{ac} & C{V}_{M}^{ac} & {\hat{\mu }}_{M}^{tr} & C{V}_{M}^{tr}\end{array}],{\boldsymbol{A}}\in {{\mathbb{R}}}^{M\times 4}\\ {\boldsymbol{C}}=[\begin{array}{c}{c}_{1}\\ \vdots \\ {c}_{i}\\ \vdots \\ {c}_{M}\end{array}],{c}_{i}=[{s}_{1}^{i},\ldots ,{s}_{14}^{i}],{\boldsymbol{C}}\in {{\mathbb{R}}}^{M\times 14}\end{array}$$

where the *i*^th^ row of matrix ***A*** encodes the four activity metrics, $({\hat{\mu }}_{i}^{ac},C{V}_{i}^{ac},{\hat{\mu }}_{i}^{ac},C{V}_{i}^{tr})$, of participant *i* and the *i*^th^ row of matrix ***C*** encodes the fourteen clinical assessment results, $({s}_{1}^{i},\ldots ,{s}_{14}^{i})$, of participant *i*. There are *M* = 13 participants in total. Furthermore, to assess the importance of individual correlations, a significance level of *α* = 0.05 was employed.

Preprocessing and calculation of activity measures were done using the Python programming language version 3.6 (Python Software Foundation). Correlations and their significance were calculated using the R programming language version 3.5.1 (R Foundation for Statistical Computing, Vienna, Austria). Graphical illustrations and plots were created using both above-mentioned programming languages as well as Blender version 2.79 (Blender Institute, Amsterdam, Netherlands).

## Data Availability

Data and code regarding the obtained results may be obtained upon request.
